# Career progression for autistic people: A scoping review

**DOI:** 10.1177/13623613241236110

**Published:** 2024-03-13

**Authors:** Jade Davies, Anna Melissa Romualdez, Elizabeth Pellicano, Anna Remington

**Affiliations:** 1 Centre for Research in Autism and Education (CRAE), Department of Psychology and Human Development, IOE, UCL’s Faculty of Education and Society, UK; 2 Clinical, Educational and Health Psychology, University College London, UK

**Keywords:** autism, career progression, career success, employment, scoping review

## Abstract

Many autistic people are unemployed. Of those who *are* employed, many are in roles that do not reflect their skills, qualifications and/or capabilities, and little is known about how autistic people progress throughout their careers. This study aimed to review and synthesise the existing evidence about career progression for autistic people. In total, 33 studies met the criteria for inclusion, though no study directly aimed to explore the topic. Our findings suggest that underemployment is common within the autistic population. Indirectly, we identified several potential barriers and facilitators of career progression for autistic people. Possible barriers included personal (e.g. gaps in education and employment history), relational (e.g. disclosing an autism diagnosis) and organisational factors (e.g. inadequate employment support). Adequate employment support was the most frequently discussed facilitator. Future research should seek to identify the most successful employment supports for autistic people over the long term to ensure that all autistic people are able to live – and work – in ways that are meaningful to them.

**Lay abstract:**

Lots of autistic people are unemployed. Even when they are employed, autistic people might be given fewer opportunities than non-autistic people to progress in their careers. For example, assumptions about autistic people’s differences in social communication might mean they are not given as many promotions. Indeed, we know that many autistic people are in jobs lower than their abilities (known as ‘underemployment’). We reviewed 33 studies that tell us something about career progression for autistic people. Our review found that lots of autistic people want to progress in their careers, but there are many barriers in their way. For example, when they told their employer about being autistic, some people were given fewer opportunities. Research has also shown that autistic people do not get enough support to progress and that gaps in their employment history can make it difficult to progress. Our review suggested that good employment support (e.g. mentors) might help autistic people to progress in their careers. However, not much research has evaluated employment support for autistic people, which means we do not know how useful it is. Future research should find the best support that allows autistic people to live and work in ways that are meaningful to them.

Autistic people across the globe are facing an employment crisis. In the United Kingdom (UK), it is estimated that only 29% of autistic people are employed, fewer than most other disability groups (Office for National Statistics, 2022). Such strikingly low estimates are also found in the United States (US), where approximately 38% of autistic adults hold paid employment (Roux et al., 2017), and in Australia, where 38% of autistic adults are considered a part of the labour force (Australian Bureau of Statistics, 2019). These figures may understate the actual number of autistic people in employment, given the significant barriers to diagnosis that exist, and the number of autistic people who do not disclose their diagnosis at work (Aylward et al., 2021; Romualdez, Heasman, et al., 2021; Romualdez, Walker, et al., 2021; Wiggins et al., 2020). Nonetheless, there remains a considerable gap between the number of autistic people who want to work, and those who obtain employment (Hendricks, 2010; National Autistic Society, 2016).

Research in the general population suggests the cost of unemployment for mental health is vast (Milner et al., 2013, 2014; Øverland, 2016) and emerging evidence from the COVID-19 pandemic suggests job loss and unemployment are associated with decreased mental health for autistic people (Goldfarb et al., 2022; Taylor et al., 2022). It is therefore unsurprising that a growing body of research has sought to address the autism-employment gap. Indeed, an umbrella review of research on vocational interventions for autistic people identified 31 relevant systematic reviews and meta-analyses encompassing 287 primary studies that examined interventions to support autistic people in gaining and maintaining employment (Tincani et al., 2023).

While increasing the employment rate for autistic people is an important goal, entering the workforce is only one phase of employment. In a recent priority-setting exercise, autistic people identified a need for more research across the entire employment journey, with one participant asking, ‘when is there going to be an emphasis on careers for people with autism?’ (Davies, Romualdez, et al., 2023, p. 6). This concern is perhaps unsurprising given that evidence points towards the *under*employment of autistic people (Baldwin et al., 2014; Harvery et al., 2021). That is, many autistic people who have obtained work are employed in positions that do not reflect their skills, qualifications and/or wider capabilities. As such, there is a need to consider not only employment rates but also the nature of autistic people’s career trajectories across the lifespan.

Career progression (or ‘career success’) refers to the accomplishment of desired work-related outcomes and can be measured by both objective (e.g. upward mobility, greater status and/or responsibility, higher pay) and subjective criteria (e.g. increased personal fulfilment, better meeting of personal needs and preferences) (Guan et al., 2019; Spurk et al., 2018). In the general population, career progression is considered important for job satisfaction and organisational commitment (Vieira et al., 2023). Given its perceived importance, researchers have attempted to outline the process through which employees achieve career progression. For example, boundaryless career theory purports that career progression involves three key competencies: (1) knowing-why (introspection regarding why one wants to progress); (2) knowing-how (human capital – development of relevant skills and expertise) and (3) knowing-whom (social capital – development of professional connections and organisational reputation) (Arthur et al., 1995, 2017). Evidence supports the association between the ‘three ways of knowing’ and career progression. van den Born and van Witteloostuijn (2013) found the three ways of knowing were associated with objective and subjective measures of career success for 1,612 freelancers in the Netherlands, while qualitative research with senior academics highlighted the three ways of knowing as key contributors to career success in academia (Beigi et al., 2018).

Autistic people, however, may face limited opportunities to acquire the outlined competencies. For example, autistic people are likely to face distinctive challenges in developing human capital (knowing-how). Human capital relates to the education, skills and expertise one possesses relevant to the job role and can be measured by one’s highest level of education, level of training and years of relevant employment experience (Baron, 2011). However, autistic people face significant barriers during their educational and employment journeys. First, many autistic children experience gaps in their primary and secondary (aged 5–16 years) education due to inappropriate learning environments, a lack of adjustments, insufficient understanding of autism among staff, limited services and a lack of specialist school placements for academically able autistic students (Bailey & Baker, 2020; Parsons & Lewis, 2010). Such poor school experiences may discourage some autistic people from pursuing higher education. Those who do attend university report similar barriers, and perhaps consequently, autistic university students are at a high risk of ‘dropping out’ (Cage & Howes, 2020; Cage & McManemy, 2022; Irvine & MacLeod, 2021). As a result, autistic people may have a lower overall level of education than non-autistic people, despite having similar capabilities.

Moreover, autistic people face a series of barriers to securing employment (Harmuth et al., 2018; López & Keenan, 2014; Scott et al., 2018), which may mean they possess fewer years of relevant employment experience than non-autistic people. Even when able to secure employment, autistic people report facing significant barriers, including inaccessible working environments and challenges associated with managing workplace demands and expectations, which can negatively impact their mental health, and increase the risk of burnout (Davies et al., under review; Tomczak & Kulikowski, 2024). These barriers compel some autistic employees to pursue more casual, part-time or precarious work arrangements (Baldwin et al., 2014; Davies et al., 2022; Pellicano et al., 2023). Together, these education- and employment-related barriers are likely to impact autistic people’s opportunities to develop the human capital that supports career progression.

Autistic people may also face limited opportunities to develop the social capital (knowing-whom) required to progress in their careers. Social capital relates to one’s professional connections and is often measured by the estimated size, quality and perceived value of one’s professional network, as well as one’s time spent networking (Avci, 2020; Wolff & Moser, 2006, 2009). Yet, differences in communication styles between autistic and non-autistic people may result in mutual misunderstandings, making social aspects of employment more challenging (Camus et al., 2022; Milton et al., 2022; Milton, 2012). Similarly, autistic people, particularly those with marginalised intersectional identities (e.g. autistic and non-White), are likely to face significant stigma and discrimination in the workplace (Doyle et al., 2022; Johnson & Joshi, 2016), creating further barriers to successful relationship building in the workplace.

As outlined, autistic people’s opportunities are likely to be constrained with regard to successful career progression. We do not, however, have a full understanding of the relevant issues, given that this topic has not been systematically examined by researchers. We therefore conducted a scoping review of the extant literature to examine what is already known about autistic people’s experiences of career progression. Specifically, we aimed to find out what the existing literature tells us about (1) autistic people’s views and experiences of career progression, (2) potential barriers to successful career progression for autistic people and (3) potential facilitators of successful career progression for autistic people. A scoping review allows researchers to map an area of literature to explore the extent and nature of existing research, summarise key research findings and identify potential knowledge gaps (Arksey & O’Malley, 2007; Levac et al., 2010). Unlike systematic reviews, scoping reviews do not attempt to provide a comprehensive synthesis of existing material, but instead provide a broad, top-level overview of research on a particular topic (Davis et al., 2009; Peters et al., 2015). A scoping review was considered most appropriate here, given the expected dearth of literature on autistic people’s experiences of career progression.

## Method

We followed the five stages as set out by Arksey and O’Malley (2007), including developing a review question, identifying relevant studies, selecting manuscripts for inclusion, extracting relevant data and summarising the results.

### Search strategy

We conducted a comprehensive search of the following databases: PsycINFO, Scopus, Web of Science, EBSCO, Proquest Dissertations & Theses and Proquest Preprints. Using previous reviews on autism and employment (e.g. Harmuth et al., 2018; Scott et al., 2018), and guidance from a specialist university librarian, we developed a search string for the current review. Initial iterations of the search string were piloted on all databases until a search string that captured the most eligible studies was found. The final string searched for the terms (autis* OR asperger* OR ‘pervasive developmental disorder’ OR pdd OR asd OR neurodivers*) AND (employment OR career OR workplace) AND (success OR progress* OR advance* OR underemploy*) in the title, abstract and keywords fields. An initial search was conducted in July 2022, and a final search in April 2023. As we did not expect career progression to be the focus topic of many studies, we conducted an additional Google Scholar search to identify studies that contained the terms ‘autism’ and ‘career progression’ or ‘career advancement’ anywhere in the article. Specifically, we screened the first ~1,000 entries from a Google Scholar advanced search, in line with existing guidance (Haddaway et al., 2015).

### Inclusion and exclusion criteria

We included studies that reported data on (1) autistic people’s experiences of career progression from the perspectives of autistic people (including formally diagnosed and self-diagnosed autistic people), their families (e.g. parents/caregivers) and/or work or school staff (e.g. managers, colleagues, teachers); (2) career success for autistic people (including both objective and subjective measures) or (3) the matching between autistic people’s capabilities and their employment, even if these data were not the focus of the research being conducted. Quantitative, qualitative and mixed-method studies were included. Studies that explored disabled people’s employment experiences more broadly were included only if data specific to autistic participants could be isolated and extracted. Doctoral dissertations/theses are typically peer-reviewed and are expected to be of publishable standard. As such, we made the decision to include them in this review. All other grey literature, including dissertations at all other levels, were excluded. Searches were limited to articles published in English and there were no restrictions on publication date.

### Study selection

**Figure 1.**
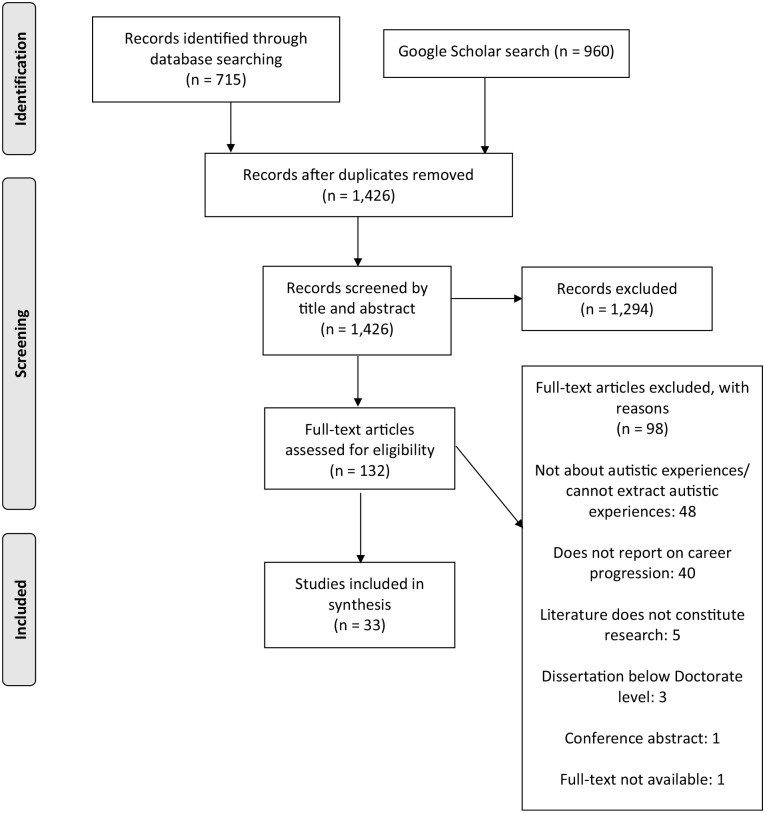
PRISMA diagram.

Search results were imported into Endnote for screening, and duplicates were removed. Two researchers (J.D. & A.P.H.) independently screened potentially eligible titles and abstracts, with reference to the inclusion/exclusion criteria (96% agreement). Discrepancies were resolved through discussion. In total, 118 studies were eligible for the full-text review. The full-text review was conducted by three researchers, with one researcher (J.D.) reviewing all articles and the remaining two (a visiting student at University College London & A.M.R.) reviewing 79 and 39 articles, respectively. Interrater agreement at this stage was 90% and 78%, respectively, and discrepancies were resolved through discussion (see Figure 1 for PRISMA flow diagram).

### Data extraction

One researcher (J.D.) independently extracted data from the included studies using a bespoke extraction form, created using Microsoft Excel. Data extracted included (1) the country (or countries) the research was conducted in, (2) study design, (3) sample characteristics (including sample size, age, gender, ethnicity, other diagnoses, highest level of education, employment status, employment sector, income), (4) study description and (5) key findings relating to career progression. Another researcher (a visiting student at University College London) checked the completed data extraction form against all included articles to ensure accuracy and completeness.

### Data synthesis

Given the number of qualitative and mixed-methods studies included in the review, a textual narrative synthesis was considered the most appropriate approach (Lucas et al., 2007). This process involved the lead author (J.D.) familiarising herself with each study through reading and rereading. Next, data perceived to be relevant to the review were extracted verbatim into the data extraction form. To be included, it had to be clear that the data were not an interpretation of the author(s), but rather came directly from participants. Extracted data were organised by the three a priori aims of the review, which included identifying (1) autistic people’s views and experiences of career progression, (2) potential barriers to successful career progression for autistic people and (3) potential facilitators of successful career progression for autistic people. Sub-themes under each research topic were inductively generated by grouping extracted data that were perceived to be conceptually similar and assigning appropriate summary headings. We took a semantic approach, referring to the explicit meanings presented in the text rather than making assumptions about underlying meanings, to ensure the fidelity of the synthesis to the participants’ perspectives. The synthesis was conducted by the first author (J.D.), with support from all three co-authors (A.M.R., E.P. & A.R.). The final synthesis was agreed upon by all authors.

### Critical appraisal

We assessed the quality of eligible studies using the Mixed Methods Appraisal Tool (MMAT) (Hong et al., 2018). To be included in the review, studies had to meet two screening criteria pertaining to the clarity of the research questions and the ability of the collected data to address the said questions. No studies were excluded on this basis. The included studies were then assessed in terms of five quality criteria, which differed according to study design. Two authors (J.D. & A.M.R.) independently assessed each study, except for one study which was part of A.M.R.’s Doctoral thesis (Romualdez, 2021) and was reviewed by J.D. and E.P. Agreement was excellent, at 90% and 93%, respectively. Discrepancies were resolved via discussion.

### Community involvement

Autistic community members or researchers were not involved in any aspect of this review.

## Results

### Descriptive summary of included studies

In total, 33 studies were included in the review (see Figure 1). Of the included studies, 22 (66.7%) used qualitative research methods, 3 (9.1%) used quantitative research methods and 8 (24.2%) used a combination of methods. The studies were conducted in the US (*n* = 15), UK (*n* = 10) and Australia (*n* = 6). Two studies (Cheriyan et al., 2021; Dreaver et al., 2020) included participants from multiple countries, including Australia, the UK, the US, Sweden and six undisclosed countries. The publication year of the included studies ranged from 2004 to 2023, with the topic receiving more attention over time: 73% (*n* = 24) of the articles were published between 2019 and 2023.

The included studies involved a total of 1,468 autistic people, 156 parents of autistic people, 96 employers of autistic people and 61 other professionals who worked with autistic people (e.g. educators, job mentors). No study reported participants fitting into multiple categories (e.g. autistic parents of autistic people). In total, 1,640 autistic people were represented, via their own input (*n* = 30 studies) and/or proxy reports (*n* = 14 studies). As seen in Table 1, almost half of the autistic people represented were female (*n* = 660 of 1,401; 47.1%) and educated to a university degree level or above (*n* = 378 of 685, 55.2%). Over three-quarters (*n* = 680 of 870; 78.2%) were from a White ethnic background and employed at the time of the study (*n* = 783 of 1,018; 76.9%). Very few were reported to have an intellectual disability (*n* = 26 of 440; 5.9%).

**Table 1.** Characteristics of the autistic people represented in the 33 studies included in the review.^a,b^.

Demographics	No. of studies reporting relevant information	Categories	Value
Gender	29 (*n* = 1,401)	Male	676 (48.3%)
		Female	660 (47.1%)
		Other gender identities	65 (4.6%)
Age (in years)	22 (*n* = 844)	Range	18–80
		Median of the mean	29.5
Ethnicity	14 (*n* = 870)	White	680 (78.2%)
		Other ethnic group (e.g., Asian, Black, Hispanic, mixed)	190 (21.8%)
Intellectual disability	11 (*n* = 440)	No	414 (94.1%)
		Yes	26 (5.9%)
Common co-diagnoses^c^	9 (*n* = 500)	Mental health condition (unspecified)	168 (33.6%)
		Anxiety	135 (27.0%)
		Depression	119 (23.8%)
		Physical health issue	80 (16.0%)
		ADHD	32 (6.4%)
Highest level of qualifications	12 (*n* = 685)	University qualifications or above	378 (55.2%)
		Vocational qualifications (e.g. a certificate, diploma, associate’s degree, vocational education)	200 (29.2%)
		Non-vocational qualifications, below university level	107 (15.6%)
		No formal qualifications	
Employment status	23 (*n* = 1,018)	Employed (including supported employment, self-employment)	783 (76.9%)
		Student/currently in training	110 (10.8%)
		Unemployed	80 (7.9%)
		Volunteer	12 (1.2%)
		Retired	5 (0.5%)
		Other	28 (2.8%)
Employment hours	9 (*n* = 463)	Full-time employed	198 (42.8%)
		Part-time/casually employed	265 (57.2%)
Level of occupation	13 (*n* = 475)	Level 1 or 2 (i.e. elementary, and entry-level occupations – basic education and/or on-the-job training required)	219 (46.1%)
		Level 3 or 4 (i.e. professional, and associate professional occupations – significant education/training required)	256 (53.4%)
Income	6 (*n* = 629)	Please see Supplementary Table 2 for details	

a Numbers and percentages may not add up to the total due to missing data/rounding.

b This table contains data regarding all autistic people represented (i.e. data provided by both autistic people and proxy reporters). Proxy-reported data only covered gender, age, employment status and level of occupation.

c For a full list of all reported co-diagnoses, see Supplementary Table 1. Co-diagnoses percentages do not add up to 100% as they were not mutually exclusive.

### Critical appraisal

The MMAT discourages the calculation of a ‘quality score’ for each study, instead advocating for a description of how studies do and/or do not meet the criteria (see Supplementary Table 3). The overall quality of the included studies was sound, particularly for qualitative studies. Common issues centred around inadequate reporting, including, for example (1) not capturing or reporting key demographic information, such as ethnicity, education or age; (2) failing to gain a representative sample of autistic participants; (3) not providing sound reasoning for the methodology used; (4) failing to provide key information about how the study was conducted, and/or (5) having missing outcome data. The quality of the studies was considered when interpreting and reporting the findings.

### Narrative summary of included studies

We performed a textual thematic synthesis on eligible studies. Data were organised according to our review questions, including: (1) what does existing literature tells us about autistic people’s views and experiences of career progression, (2) what does existing literature tells us about possible barriers to career progression for autistic people and (3) what does existing literature tells us about possible facilitators of career progression for autistic people. In total, 11 sub-themes were inductively generated under each review question. A summary of the review findings can be found below, and in Table 2 (see also Supplementary Table 4 for full details of included papers). We prioritise reporting studies that include direct reports from autistic people themselves.

**Table 2.** Summary of review findings.

Theme	Relevant studies *n* (%)
Autistic people’s views and experiences of career progression (*n* = 7, 21.2%)	
1.1 Career progression is desirable to many (but not all) autistic people	7 (100%)
Possible barriers to career progression for autistic people (*n* = 25, 75.8%)	
2.1 Underemployment and poor job-matching	16 (64.0%)
2.2 Systemic stigma, discrimination and oppression	9 (36.0%)
2.3 Organisational barriers	4 (16.0%)
2.4 Gaps in education and employment histories	3 (12.0%)
2.5 Differences in social communication	3 (12.0%)
2.6 Health issues associated with employment	2 (8.0%)
2.7 Difficulties setting career goals	2 (8.0%)
2.8 Financial implications of career progression	1 (4.0%)
Possible facilitators of career progression for autistic people (*n* = 13, 39.4%)	
3.1 Adequate employment support	11 (84.6%)
3.2 Tailored opportunities for career progression	2 (15.4%)

### Review question 1: autistic people’s views and experiences of career progression

#### Career progression is desirable to many (but not all) autistic people

Seven studies referenced the desirability of career progression for autistic people. Most (*n* = 6, 86%) indicated autistic people want to progress in their careers. For example, Hayward et al. (2019) reported that many of their participants desired ‘fulfilling’ careers with opportunities for growth and development. When asked to reflect on what successful employment would look like for them, Raymaker et al.’s (2023) autistic participants highlighted the importance of ‘opportunities for professional growth’ (p. 71). Autistic participants working under 40 hours per week in Coleman and Adams’s (2018) study said they would like to work more hours, while Cheriyan et al.’s (2021) participants reported that their top goal in attending university was to improve their career prospects. Similarly, when asked why they signed up to receive workplace mentoring, Buckley et al.’s (2022) participants expressed hopes for support in progressing in their careers. Finally, the mother of an autistic person in Braudis’s (2017) study reported that her son was not interested in the ‘lower functioning jobs that some people want to put him in’ (p. 51). Only one study indicated that career progression may not be desirable for some autistic people. In Anderson and colleagues’ (2021) study, some autistic people were characterised – by their parents – as being comfortable in their current job role and did not prioritise career progression: ‘[Elena] is not interested in seeking a higher paying job’ (p. 97).

### Review question 2: possible barriers to career progression for autistic people

#### Underemployment and poor job-matching

In total, 13 studies discussed the underemployment of autistic people. Of those, five studies attempted to quantify underemployment in their sample: 46% of Baldwin et al.’s (2014) sample (*n* = 130, all without co-occurring intellectual disability) were considered ‘overeducated’ for their current role; 42% of Braudis’s (2017) interviewees (*n* = 12) expressed concerns about being underemployed; 11% of Coleman and Adams’s (2018) sample (*n* = 74) expressed a need for better job placement and 8% talked about being placed in jobs below their ability level; 37% of Harvery et al.’s (2021) non-representative sample (*n* = 149) were considered underemployed, and 44% of Sharpe et al.’s (2022) participants (*n* = 9) had jobs that were not relevant to their career aspirations. In eight studies, participants provided qualitative evidence of the underemployment autistic people face. For example, one of Hayward and colleagues’ (2019) female participants shared: ‘I’m 52, [I have] two degrees, [but] haven’t worked a real job since 1997 unless you count $20/hour dogsbody stuff’ (p. 302). This sentiment was echoed by Hurlbutt and Chalmers (2004) and Elichaoff’s (2015) participants. Women in North’s (2023) study shared similar experiences, explaining they often had ‘much better qualifications’ (p. 14) than required. Similarly, Hedley et al.’s (2021) participants also reported their ‘frustration’ at being stuck in low-paid work with little meaning and Hayward et al.’s (2018) participants reflected on the instability they often experienced in employment, reflecting they had to take ‘whatever [job] is available’ (p. 54). In two studies, parents and employers discussed the underemployment autistic people face: parents of autistic people in Anderson et al.’s (2021) study reported concerns about their children working below their ability, while employers in Nicholas and Lau’s (2019) study identified the underrepresentation of autistic people in managerial roles within their organisation.

Four studies also reported on poor job-matching by job coaches and/or government employment support programmes, which resulted in underemployment, and hindered opportunities for career progression. Raymaker et al.’s (2023) participants reported that government-funded employment support programmes for people with disabilities were unable to connect autistic candidates to appropriately skilled positions. One of Sharpe and colleagues’ (2022) participants had been placed in the fast-food sector despite expressing an interest in transport, while one of Berman’s (2022) participants was given a job as a ‘dog pooper scooper’ despite having a Bachelor’s degree. Ortiz’s (2018) participants shared similar experiences:

She just acquired her Masters, she has done social work, and they want to put her in a factory doing Velcro. . . there is nothing wrong with doing the Velcro, if that’s the fit for you, but agencies that are partnering need to be thinking about individualizing things. (p. 93)

#### Systemic stigma, discrimination and oppression

Nine studies suggested that autistic people may be denied career progression opportunities by virtue of their autistic identity and/or traits. Elichaoff’s (2015) participants put their lack of career progression down to being autistic, often without knowing until later in life: ‘I’ve got three degrees (two undergraduate, one PhD), and I got no career, and the reason for that is, now I know, is because I’ve got Asperger’s Syndrome’ (p. 1858). Yet, a participant in North’s (2023) study felt an earlier diagnosis may have reduced their potential for success: ‘If I had been diagnosed as a child and known that only 13% of autistic people actually hold employment, maybe I wouldn’t have been able to do [myself] credit’ (p. 13). One study identified an association between autistic traits and underemployment. Specifically, Harvery et al. (2021) found that autistic people with fewer autistic traits, as indexed by the Autism Spectrum Quotient Short Form (Hoekstra et al., 2011), were more likely to be underutilised (*p* = .003) and underemployed (*p* = .018). The model remained significant when excluding self-identified participants, and those with intellectual disability.

Participants in six studies shared concerns about the potential negative impact of disclosure on their career success. For example, Buckley et al.’s (2021) participants were concerned about disclosure resulting in being ‘pigeon-holed’ into autism-specific work, thus limiting their work opportunities. Hedley et al.’s (2021) participants also shared concerns about the negative impact of disclosure on ‘job prospects’, while one participant in Romualdez’s (2021) non-representative sample feared disclosure may ‘be used as an excuse not to promote me or give me a pay rise’ (p. 59). Participants in three further studies shared qualitative evidence of reduced opportunities for progression following disclosure. For example, one of Wood and Happé’s (2021) survey participants reported being ‘sacked’ after receiving their diagnosis, while another was advised not to disclose, which they felt left their career ‘in ruins’ (p. 12). One of Harvery et al.’s (2021) participants also reported being turned down from an internal opportunity after disclosing, while one of Djela’s (2021) participants shared: ‘my openly autistic acquaintance is treated differently from me and given less opportunities than me’ (p. 90).

#### Organisational barriers

Four studies indicated that organisations are often inadequately equipped to support their autistic colleagues, which created particular barriers related to career progression. For example, Hayward et al. (2019) reported that inadequate organisational support had left their participants with ‘low hope for finding meaningful employment’ (p. 302). Similarly, one of Balubaid’s (2017) participants said: ‘There’s not enough done to help us. . .we’ve got skills and talents. . .to give to the world but we don’t know how to go on to do the next stage or progress our career’ (p. 107). Berman’s (2022) participants highlighted the lack of understanding within organisations and suggested the lack of support could be a particular problem for those considered cognitively able, with one participant being told ‘[you’re] too high functioning, you don’t quality [for employment support], you don’t need it’ (p. 159). Finally, North’s (2023) participants reported that internal promotion processes, such as interviews, acted as a key barrier to career progression for autistic people.

#### Gaps in education and employment histories

Three studies suggested that gaps in education and employment histories may act as a barrier to career progression for autistic people. Indeed, Sharpe et al.’s (2022) participants reported lacking confidence in their ability to achieve their aspirations, feeling as though a lack of skills was holding them back. Similarly, an autistic academic in Sang and colleagues’ (2022) study explained they were unable to work the same number of hours as their colleagues, resulting in a ‘sparser publication record’ (p. 9), something they felt would ‘inevitably’ negatively impact their career. Parents in Anderson et al.’s (2021) study also spoke of their challenges in helping their child(ren) secure relevant ‘career-building’ experience, which was perceived as a barrier to meaningful employment. In one case, an autistic person ‘gave up his job search to enrol in community college’ (p. 97) due to their lack of experience.

#### Differences in social communication

Three studies suggested that differences in social communication may hinder autistic people’s career progression. For example, Buckley et al.’s (2021) participants discussed the importance of professional networking in the creative arts industry and reported feeling disadvantaged in this respect. Discussing the need to take on managerial responsibilities in higher-level positions, one of Cockayne’s (2019) participants said, ‘I couldn’t manage people. . .I’m too direct. . .maybe management would not be my forte’ (p. 198). This sentiment was shared by Braudis’s (2017) participants, with one participant explaining ‘to get from the entry level to the upper level there are all these social niceties and all these politics of how you get there, not necessarily skills individuals with ASD are adept. It results in underemployment’ (p. 59).

#### Health issues associated with employment

Two studies highlighted health issues, including burnout, as a factor negatively impacting career progression for autistic people. Autistic school staff in Wood and Happé’s (2021) study reported experiencing significant mental health issues that they felt were due to their negative workplace experiences. For one participant, this resulted in burnout, and ultimately taking a demotion. Raymaker et al.’s (2023) participants also suggested that burnout may be a key contributor to a lack of long-term employment success for autistic people.

#### Difficulties setting ‘realistic’ career goals

Two studies suggested that autistic people may experience difficulties in setting appropriate career goals, which may hinder their career progression. In Wong and colleagues’ (2020) study, school personnel who were involved in employment preparation for autistic students reported that many autistic people experienced challenges in setting ‘realistic and practical’ career goals. Similarly, two of Braudis’s (2017) non-autistic interviewees said some autistic people have ‘unrealistic’ career expectations, including ‘expecting top-level positions. . .and having specific and unwavering career goals’ (pp. 57–58).

#### Financial implications of career progression

Just one participant in one study indicated that the financial consequences of career progression may act as a barrier for autistic people. Lindstrom et al. (2014) examined the career advancement experiences of four people with intellectual and developmental disabilities over 9 years. One of the case studies (Rick) was autistic. Rick’s advocate explained that he was unable to take on more hours at work as this would negatively impact the benefits he was eligible to receive. As such, he was limited to part-time industry work, something the authors suggested resulted in reduced opportunities for progression. Indeed, Rick stayed on minimum wage for the entire study, and was US$2,200 below the federal poverty line in the 9-year follow-up.

### Review question 3: possible facilitators of career progression for autistic people

#### Adequate employment support

In total, 11 studies indicated that adequate employment support (including both formal and informal support) may facilitate career progression for autistic people. Autistic people in the creative arts discussed a desire for an employment mentor with whom they could consult about workplace challenges and receive advice regarding career progression (Buckley et al., 2021). Similarly, Hurlbutt and Chalmers’s (2004) participants reflected on the fact they had not received any employment support, but endorsed the idea, noting that ‘one person can make a difference in how successful the person with autism will be’ (p. 219). Evidence from studies in which participants *had* received support followed this sentiment. For example, the use of an employment mentor was perceived to have contributed to employment success for one of Braudis’s (2017) case studies and Berman’s (2022) study indicated that participants who received individualised employment support were successful in achieving their employment goals.

Seven studies evaluated the impact of formal sources of employment support, such as employment interventions. Of those, two studies evaluated the use of Project SEARCH plus Autism Spectrum Disorder Supports (Project SEARCH + ASD).^1^ Wehman et al. (2017) tested the effectiveness of Project SEARCH + ASD using a randomised controlled trial. Participants receiving the intervention worked significantly more hours (*p* < .001) and earned significantly higher wages (*p* < .001) than those not receiving the intervention, at the 3-month follow-up. Participants receiving the intervention also worked significantly more hours (*p* < .001) than those not receiving Project SEARCH + ASD at the 12-month follow-up, though there was no significant difference in wages. None of these differences were maintained, however, when unemployed participants were removed from the analysis. One of Ham et al.’s (2014) participants reportedly had an increase in working hours after receiving the support outlined in their ‘behaviour intervention and work productivity’ plan, though it is unclear how this information was gathered.

Five studies evaluated alternative initiatives. McLaren et al. (2017) evaluated a pilot of Individual Placement and Support (IPS)^2^ with five autistic people (80% male, all with ‘moderate’ or ‘severe’ needs, as indexed by the Social Responsiveness Scale, Second Edition) (Constantino & Gruber, 2012). The researchers reported that, following the pilot, all clients had received an increase in pay and work hours compared with their previous employment. Brooke et al. (2018) evaluated the success of an employment support service over an 18-month period. Over the 18 months, most participants (*n* = 61 of 70; 87%) retained employment; of those, four (7%) were reported as advancing in their employment. Buckley et al. (2022) conducted an evaluation of a mentoring programme for autistic people in the creative arts and found that the programme created new employment opportunities and supported attendees in mapping out career strategies. In their qualitative research, Nicholas and Lau (2019) evaluated employment support provided for autistic people via a specialised organisation providing support to autistic people in the information technology sector. Support providers spoke of their work in ‘giving people opportunities to succeed’ (p. 198) and reported that when well-supported, the autistic consultants were able to ‘thrive’. Similar findings were identified by Grenawalt et al. (2020) whose participants discussed how their autism-employment initiative provided unique opportunities for advancement.

Regarding less formal sources of support, Harvery and colleagues (2021) found that the size of participants’ overall support networks predicted their odds of being appropriately employed (*p* = .015) but not being underemployed (*p* = .07). The authors also found that autistic participants receiving workplace adjustments were 3.14 times more likely to be in a job adequately matched to their skill level (*p* = .035), though this was not sustained when excluding self-identified autistic participants (*p* = .134).

#### Tailored opportunities for career progression

Two studies involving employers of autistic people indicated a need to tailor opportunities for career progression to autistic people’s individual needs. Dreaver et al. (2020) examined the perceived factors associated with successful employment for autistic people, from the perspective of employers. Participants discussed the need to ‘slowly build up the work role’ and ‘figure out how to challenge’ autistic employees (p. 1663), as a way of providing suitable opportunities for career progression. Similarly, Nicholas and Lau’s (2019) participants highlighted the need to match opportunities for career progression to autistic people’s individual career goals and support needs.

## Discussion

In this scoping review, we synthesised the extant literature on career progression for autistic people. Notably, we were unable to identify any studies that directly focused on career progression. Most of the eligible studies examined employment experiences more broadly, with data that were perceived as relevant to this review being identified and extracted by the reviewers. What remains lacking, therefore, is a focused exploration of career progression for autistic people, directly examining autistic people’s experiences of progressing throughout their career, as well as their perceptions regarding the barriers to, and facilitators of, career progression. In what follows, we reflect on our key findings, considering how they map on to existing frameworks of career progression, and provide key suggestions for future research and practice.

Our review suggests that, while many autistic people desire career progression, they are often underemployed, ‘stuck’ in poorly matched job roles and receive few opportunities and little support to progress in their careers. Several studies included in this review attempted to quantify underemployment in their samples, with estimates suggesting up to 46% of autistic people may be employed in jobs below their capability and/or capacity (Baldwin et al., 2014). Our findings suggest that such underemployment may be exacerbated by external agencies (e.g. disability employment providers) who are motivated to place autistic people in the first job that arises, as opposed to the job that is the most appropriate fit to the individual’s preferences, skills and abilities (Berman, 2022; Ortiz, 2018; Raymaker et al., 2023; Sharpe et al., 2022). This underutilisation of autistic talent is problematic for several reasons. First, underemployment has negative implications for people’s mental and physical health, and the impact of underemployment on mental health is thought to be more pronounced for disabled people (Allan et al., 2022; Friedland & Price, 2003; Milner et al., 2017; Milner & Lamontagne, 2017). This is particularly alarming given that autistic people are already considered more vulnerable to poor health outcomes (Cashin et al., 2016; Croen et al., 2015; Lai et al., 2019). Second, underemployment comes at a significant economic cost to individuals, organisations and society more broadly (Barnichon & Zylberberg, 2019; Lloyd-Cape, 2020). As such, reducing underemployment for autistic people should be considered a key target outcome for future research and practice. To address the issue of underemployment, we must understand its underlying causes. The findings of this review provide important insight into the possible barriers to, and facilitators of, appropriate employment and career progression for autistic people. Next, we map the identified barriers and facilitators on to the three key competencies outlined by Arthur and colleagues (1995, 2017).

### Competency 1: knowing-why

Some non-autistic people (e.g. educators, employers) included in this review raised concerns about autistic people’s ‘unrealistic’ career expectations (Braudis, 2017; Wong et al., 2020). However, it is also possible that other people’s (low) expectations of autistic people are a barrier here. Indeed, low presumptions of competence – which may be perpetuated by deficit-based models of autism (Biklen, 2020) – were observed within several of the sub-themes identified in this review, including the way that autistic people were commonly matched to roles well below their capabilities. Future research should seek to confirm autistic people’s views on setting career expectations, including whether support in this regard would be welcomed.

Nonetheless, several career planning tools have already been developed and implemented with autistic people. For example, one systematic review identified 14 studies evaluating 10 different career planning tools used with autistic people (Murray et al., 2016). Here, the authors raised concerns regarding the lack of autism-specific tools, as well as the overall utility of existing tools, noting that ‘none meet the full requirements for reliability and validity suggested for predictive tools’ (Murray et al., 2016, p. 199). Since then, several other planning tools and techniques for autistic people have been described and/or evaluated (e.g. Hatfield et al., 2017; Walsh et al., 2019). However, to our knowledge, most, if not all, tools are aimed at autistic youth who are entering the workforce for the first time, and not for those at later entry points or stages in their careers. Future research may therefore seek to establish and evaluate the career planning support available to autistic people already in employment.

### Competency 2: knowing-whom

One of the key barriers identified in relation to the knowing-whom competency was the systemic stigma, discrimination and oppression that autistic people commonly face in the workplace. Participants in the included studies were particularly concerned that disclosing an autism diagnosis would result in fewer progression opportunities (Buckley et al., 2021; Djela, 2021; Harvery et al., 2021; Hedley et al., 2021; Romualdez, 2021; Wood & Happé, 2021). Such concerns were not unfounded: some participants had experienced, or witnessed, instances in which disclosure of an autism diagnosis had resulted in the unfair termination of employment and/or reduced opportunities for career progression (Djela, 2021; Harvery et al., 2021; Wood & Happé, 2021). These findings, unfortunately, are not surprising. Autistic people consistently report facing stigma and discrimination in the workplace (Davies, Romualdez et al., 2023; Doyle et al., 2022; Johnson & Joshi, 2016). The elimination of such poor workplace experiences must be a priority for future research and practice.

One common suggestion for decreasing stigma is the provision of training that improves knowledge and understanding of autism (Davies et al., 2022; Romualdez, Heasman, et al., 2021; Turnock et al., 2022). Encouragingly, research suggests that autism-specific training programmes can increase knowledge, lower explicit biases and improve first impressions of autistic people (Gillespie-Lynch et al., 2015; Jones et al., 2021; Obeid et al., 2015; Saade et al., 2023). However, such programmes appear to be less effective at reducing implicit biases and it remains unclear whether these findings are translated into practice (Jones et al., 2021, though see Gillespie-Lynch et al., 2021). Moreover, little research has examined the effectiveness of such training programmes in employment contexts, and to our knowledge, no study has examined whether any potential improvements in knowledge and/or reductions in stigma and discrimination are sustained over the long term. As such, little is known about what works to reduce stigma and discrimination for autistic people at work. This is a key avenue for future research.

Also related to the knowing-whom competency, our findings suggest that differences in reciprocal social communication between autistic and non-autistic people (Milton, 2012) may put autistic people at an unfair disadvantage when it comes to progressing in their careers. First, our review suggests that professional networking may be particularly challenging for some autistic people (Buckley et al., 2021). Yet, traditional career success is – at least in part – contingent on one’s ability to build and leverage professional connections, meaning autistic people are likely to be at a disadvantage (Avci, 2020; Wolff & Moser, 2006, 2009). Similarly, our review findings indicate that, in the context of career progression, social adeptness may be prioritised by employers over professional competence. Indeed, some autistic people did not feel able to meet neurotypical social expectations, resulting in reduced career progression opportunities, even if they excelled professionally (e.g. Braudis, 2017). Relatedly, some participants in the included studies highlighted a paucity of opportunities for employees who may not desire, or feel capable of managing others (e.g. Cockayne, 2019). This lack of appropriate opportunities is likely to reinforce the underemployment of autistic people. One way to address this concern may be to offer more tailored, individualised opportunities for career progression (e.g. Dreaver et al., 2020; Nicholas & Lau, 2019), moving away from a rigid model that assumes managerial responsibilities are the main criteria for success and towards a more holistic framework that recognises and values diverse strengths and career aspirations. This shift could involve creating pathways that emphasise skill development, project (rather than person) leadership or other forms of professional growth that do not necessarily require traditional managerial roles.

### Competency 3: knowing-how

Participants in the included studies highlighted their perceived lack of skills and experience as a barrier to finding appropriate employment opportunities and progressing in their careers (Anderson et al., 2021; Sang et al., 2022; Sharpe et al., 2022). Existing research suggests autistic people are likely to face significant barriers in accessing employment. For example, many autistic people experience sensory differences (Proff et al., 2022; Robertson & Baron-Cohen, 2017) and/or co-occurring mental and physical health conditions (Cashin et al., 2016; Croen et al., 2015; Lai et al., 2019; Weir et al., 2021), which can make employment particularly overwhelming and unsustainable. Furthermore, common workplace social dynamics, such as unwritten social rules, ‘office politics’, and hierarchical structures, pose particular challenges for some autistic people (Diener et al., 2020; Ezerins et al., 2023; Harmuth et al., 2018; Scott et al., 2018). While workplace adjustments can address many of these barriers, their implementation is often inconsistent, and effectiveness varies (Davies et al., 2022). Consequently, many autistic people leave the workforce prematurely, or find themselves compelled into part-time or casual employment, below their capabilities (Baldwin et al., 2014; Davies et al., 2022; Pellicano et al., 2023). The prevalence of underemployment among autistic people exacerbates the challenges they face, not only limiting the opportunities for cultivating career-related skills but also resulting in a dearth of relevant employment experience compared with their non-autistic counterparts. This predicament has the potential to perpetuate a cycle of limited career development for autistic people.

We make two key recommendations to support autistic people in developing the ‘knowing-how’ competencies (i.e. the human capital) required to progress. First, employers should ensure they are aware of the systemic barriers that autistic candidates are likely to face in accessing education, employment and training, and ensure they do not inadvertently discriminate against them based on potential gaps in these areas. For example, they should reconsider using artificial intelligence within their hiring processes (e.g. curriculum vitae screeners), which typically evaluates candidates against the ‘standard’ person specification and may therefore screen out candidates that ‘appear less qualified only due to systemic denial of education and employment opportunities’ (Nugent et al., 2020, p. 10). Similarly, they may consider making adjustments to hiring processes, such as providing interview questions in advance or utilising more practical hiring processes like work trials (Davies, Heasman, et al., 2023; Maras et al., 2021), to ensure autistic people have equal access to employment. Adjustments within the workplace – such as adjustments to the physical environment (e.g. providing specialist equipment/software, quiet spaces), the job role (e.g. offering flexible working hours, working from home) and/or social and cultural practices (e.g. providing more explicit communication) – may also improve experiences, making employment more sustainable for autistic employees (Davies et al., 2022; Khalifa et al., 2019; Lindsay et al., 2019). Findings from one of the studies included in this review suggest that traditional promotion processes may also disadvantage autistic candidates (North, 2023). As such, adjustments in this regard are likely to be welcomed.

Second, we recommend that more autistic people be given access to tailored employment support. Disappointingly, the studies included in this review highlighted an overall lack of employment support, particularly for those without an intellectual disability, which was perceived to limit opportunities for progression (Balubaid, 2017; Berman, 2022; Hayward et al., 2019). Yet, the most commonly suggested facilitator was employment support. Several of the included studies described and evaluated possible interventions and supports. Such programmes commonly included elements of (1) individualised career planning and goal setting, (2) job-matching, (3) training on work-related skills and/or competencies and (4) mentoring (Berman, 2022; Braudis, 2017; Brooke et al., 2018; Buckley et al., 2022; Grenawalt et al., 2020; Ham et al., 2014; McLaren et al., 2017; Nicholas & Lau, 2019; Wehman et al., 2017). The findings of these studies were generally positive, suggesting that, with adequate support, autistic people could set and achieve meaningful employment-related goals. Nonetheless, many were relatively small in scale (e.g. 12–15 mentees) and/or focused solely on autistic young people (e.g. aged <25 years), with relatively high support needs. There was also a significant lack of longitudinal research, with the maximal follow-up period being 18 months. As such, the evidence gathered appears to be of limited generalisability, and little remains known about appropriate support for autistic people later in their career journey. It is also unclear which, if any, positive changes are sustained following employment interventions. Future research should seek to conduct more rigorous, longitudinal testing of support initiatives to identify the most effective ways of supporting autistic people to (1) maintain appropriate employment and (2) progress within employment.

### Limitations

While the findings offer preliminary insight into career progression for autistic people, our approach was limited in several key ways. First, our search may not have been exhaustive, especially since data relevant to this review were often embedded within the results sections of articles, as opposed to being the main research focus. Consequently, it is possible that some relevant research was not identified in our search and was therefore missed. Second, as we only included articles in which it was clear that the data came directly from participants, our review is necessarily limited by the data (e.g. quotes) that authors chose to report. As none of the included studies specifically focused on autistic people’s experiences of career progression, career progression-related quotes may not have been perceived, by the authors of the included articles, as relevant, and therefore not reported. Future research should intentionally examine autistic people’s experiences of career progression to contribute to a richer, more thorough, understanding of the factors impacting career progression for autistic people. Third, while our review focused on data explicitly related to career progression, autistic people’s broader workplace experiences are also likely to shape their experiences of career progression. For example, factors such as workplace culture, social interactions and adjustment practices are likely to impact career trajectories, even if the factors have not been explicitly linked. Future research and interventions must therefore be cognisant of the broader context in which autistic people navigate employment, to ensure a more comprehensive understanding and effective support mechanisms. Fourth, the limitations of the included studies inevitably extend into this review. Many of the studies did not adequately describe their sample, and of those that did, most involved non-representative samples (e.g. limited gender and/or ethnic diversity, no/very few participants with intellectual disability). It is also notable that many studies did not report the average age, or age range, of their participants, and several of the included studies solely examined the experiences of autistic youth who were entering the workforce for the first time. As such, the findings of this review will not reflect the views and experiences of all autistic people as it relates to career progression. Future research should examine a wider range of perspectives to ensure a more diverse range of voices are represented. Such research may also seek to examine how other factors and experiences (e.g. gender, mental health experiences, ethnic group) shape employment experiences and success for autistic people. Finally, relatively few studies (*n* = 10, 30.1%) reported involving autistic people in the research design, conduct, analysis and/or interpretation processes. When authors did report community involvement, this was mostly consultative (e.g. providing feedback on drafted documents), as opposed to partnership (i.e. shared decision-making) (Arnstein, 1969, 2019). Future autism-employment research must seek to meaningfully involve autistic people as equal researchers to ensure that research and employment practices are appropriately aligned with the needs and priorities of autistic people (Davies, Romualdez, et al., 2023).

## Conclusion

The present review highlights that many autistic people want to progress in their careers but are not given the support, tools or opportunities to do so. Many potential barriers that autistic people face in this regard are external in nature (e.g. poor response to disclosure, inadequate organisational support, inaccessible promotion processes). While employment support appears to be a key factor in career progression, little high-quality research has examined the most effective forms of support for autistic employees over the long term. This review identifies a growing need for more rigorous research that focuses on the real opportunities that are open to each autistic person to live – and work – in ways that are meaningful to them.

## Supplemental Material

sj-docx-1-aut-10.1177_13623613241236110 – Supplemental material for Career progression for autistic people: A scoping review Supplemental material, sj-docx-1-aut-10.1177_13623613241236110 for Career progression for autistic people: A scoping review by Jade Davies, Anna Melissa Romualdez, Elizabeth Pellicano and Anna Remington in Autism

## Notes

1. Project SEARCH + ASD is a school-to-employment programme, using principles of applied behaviour analysis, which aims to provide autistic people with the opportunity to learn work skills in a local business. The programme involves goal setting, internships and collaboration between school and adult services. Support is devised through an individualised ‘behaviour intervention and work productivity plan’.

2. Individual Placement and Support involves support with job searches, consideration of mental health, inclusion of client preferences, and individualised searches and supports.
