# Supplementary material for: Career progression for autistic people: A scoping review
Source: Autism. 2024 Mar 13;28(11):2690–706. doi: 10.1177/13623613241236110 (PMC11494842; doi:10.1177/13623613241236110)
Supplement: sj-docx-1-aut-10.1177_13623613241236110 – Supplemental material for Career progression for autistic people: A scoping review [file sj-docx-1-aut-10.1177_13623613241236110.docx]

| **Supplementary Table 1.**  *Full list of reported co-diagnoses (from 9 studies^a^, representing 500 autistic people)* | |
| --- | --- |
| **Diagnosis** | **N (%)** |
| Mental health condition (unspecified) | 168 (33.6%) |
| Anxiety | 135 (27.0%) |
| Depression | 119 (23.8%) |
| Physical health issue | 80 (16.0%) |
| Attention Deficit Hyperactivity Disorder (ADHD) | 32 (6.4%) |
| Obsessive Compulsive Disorder (OCD) | 27 (5.4%) |
| Dyspraxia | 24 (4.8%) |
| Dyslexia | 19 (3.8%) |
| Post-Traumatic Stress Disorder (PTSD) | 16 (3.2%) |
| Ehlers-Danlos Syndrome | 7 (1.4%) |
| Bipolar Disorder | 5 (1.0%) |
| Eating Disorder | 3 (0.6%) |
| Personality Disorder (unspecified) | 3 (0.6%) |
| Tourette’s Syndrome | 3 (0.6%) |
| Deaf/hearing loss | 2 (0.4%) |
| Dyscalculia | 2 (0.4%) |
| Panic Disorder | 2 (0.4%) |
| Prosopagnosia | 2 (0.4%) |
| Alexithymia | 1 (0.2%) |
| Borderline Personality Disorder | 1 (0.2%) |
| Downs Syndrome | 1 (0.2%) |
| ^a^ Anderson et al. (2020); Buckley et al. (2022); Ham et al. (2014); Harvery et al. (2021); Hayward et al. (2019); Hurlbutt & Chalmers (2004); McLaren et al. (2017); Romualdez (2020); Sang et al. (2021) | |

| **Supplementary Table 2.**  *Participants’ income breakdown (from 6 studies, representing 629 autistic people)* | |
| --- | --- |
| **Earnings per hour (reported in $, *n* = 191)** ^a^ |  |
| Range | $0 – $44 |
| Median of the reported means | $10.07 |
| **Annual salary (reported in $, *n* = 200)** ^b^ |  |
| Less than $25,000 | 56 (28.0%) |
| $25,001 – $50,000 | 100 (50.0%) |
| $50,001 – $74,999 | 38 (19.0%) |
| More than $75,000 | 6 (3.0%) |
| **Annual salary (reported in £, *n* = 238^)^** ^c^ |  |
| Less than £10,000 | 53 (22.3%) |
| £10,000 – £19,999 | 57 (23.9%) |
| £20,000 – £29,999 | 53 (22.3%) |
| £30,000 – £39,999 | 23 (9.7%) |
| £40,000 – £49,999 | 15 (6.3%) |
| £50,000 – £59,999 | 9 (3.8%) |
| £60,000 – £79,999 | 6 (2.5%) |
| £80,000 – £99,999 | 2 (0.8%) |
| £100,000 or more | 5 (2.1%) |
| Prefer not to say/missing | 15 (6.3%) |
| ^a^ Brooke et al. (2018); Coleman & Adams (2018); Lindstrom et al. (2014); McLaren et al. (2017)  ^b^ Berman (2022)  ^c^ Romualdez (2020) | |
|  | |

| **Supplementary Table 3.**  *Results of the study quality assessment using the Mixed Methods Appraisal Tool (MMAT).* | | | |
| --- | --- | --- | --- |
| **Study type** | **Quality criteria** | **N studies meeting criteria** | **Reasons studies did not meet criteria** |
| **1. Qualitative (n=30)** | 1.1 Is the qualitative approach appropriate to answer the research question? | 27 (90%) | One or more of the research questions would be better addressed using quantitative research (*n*=2), or, in mixed-methods studies, the research questions were better addressed by the quantitative portion of the data (*n* = 1). |
|  | 1.2 Are the qualitative data collection methods adequate to address the research question? | 25 (83%) | Lack of clarity regarding how data were collected (*n*=2); only one group was asked the relevant questions (*n*=1); no/inadequate justification for use of secondary data (*n*=1); relied on one single open-ended question (*n*=1). |
|  | 1.3 Are the findings adequately derived from the data? | 23 (77%) | Inappropriate choice of analysis (*n*=1), inadequate description of analysis (*n*=1), or lack of sophistication to analysis (*n*=5). |
|  | 1.4 Is the interpretation of results sufficiently substantiated by data? | 28 (93%) | No/inadequate number of quotations from participants (*n*=2) |
|  | 1.5 Is there coherence between qualitative data sources, collection, analysis and interpretation? | 28 (93%) | Links between data sources, collection, analysis and interpretation were unclear (*n* = 2). |
| **2. Quantitative randomised (*n*=2)** | 2.1. Is randomisation appropriately performed? | 2 (100%) | N/A |
|  | 2.2. Are the groups comparable at baseline? | 0 (0%) | Significant group differences in key demographic characteristics (e.g., age/gender/ethnicity/prior experience) (*n*=1) or no statistical comparisons made (*n*=1) |
|  | 2.3. Are there complete outcome data? | 1 (50%) | One group did not complete follow-up measure (*n*=1) |
|  | 2.4. Are the outcome assessors blinded to the intervention provided? | 0 (0%) | Assessors not blind to group assignment (*n*=2) |
|  | 2.5. Did the participants adhere to the assigned intervention? | 1 (50%) | Intervention not completed in specified timeframe (*n*=1) |
| **3. Quantitative non-randomised (*n*=3)** | 3.1 Are the participants representative of the target population? | 0 (0%) | Reporting of participant characteristics was insufficient to determine representativeness (*n*=2); overrepresentation of women (*n*=1). |
|  | 3.2 Are measurements appropriate regarding both the outcome and intervention (or exposure)? | 2 (67%) | Used non-standardised, overly simplistic measures (*n*=1) |
|  | 3.3 Are there complete outcome data? | 2 (67%) | >10% missing data on variable(s) of interest (*n*=1) |
|  | 3.4 Are the confounders accounted for in the design and analysis? | 3 (100%) | N/A |
| **4. Quantitative descriptive (*n*=5)** | 4.1 Is the sampling strategy relevant to address the research question? | 3 (60%) | Sampling strategies unlikely to yield representative sample (*n*=2) |
|  | 4.2 Is the sample representative of the target population? | 0 (0%) | Insufficient reporting of participant characteristics to determine representativeness (*n*=3); overrepresentation of one gender/sex (*n*=2) |
|  | 4.3 Are the measurements appropriate? | 4 (80%) | No reliability estimates reported (*n*=1) |
|  | 4.4 Is the risk of nonresponse bias low? | 4 (80%) | >10% missing data on variable(s) of interest (*n*=1) |
|  | 4.5 Is the statistical analysis appropriate to answer the research question? | 5 (100%) | N/A |
| **5. Mixed methods (n=8)** | 5.1 Is there an adequate rationale for using a mixed methods design to address the research question? | 5 (63%) | The rationale for the use of both qualitative and quantitative methods was not adequately explained (*n*=3) |
|  | 5.2 Are the different components of the study effectively integrated to answer the research question? | 6 (75%) | No clear attempt to integrate the qualitative and quantitative components of the in the results/discussion sections (*n*=2) |
|  | 5.3 Are the outputs of the integration of qualitative and quantitative components adequately interpreted? | 7 (88%) | Inadequate integration of the qualitative and quantitative components during the interpretation of the findings (*n*=1) |
|  | 5.4 Are divergences and inconsistencies between quantitative and qualitative results adequately addressed? | 5 (63%) | Divergencies and inconsistencies between the qualitative and quantitative results were not adequately discussed (*n*=3) |
|  | 5.5 Do the different components of the study adhere to the quality criteria of each tradition of the methods involved? | 2 (25%) | The overall quality of one or both components was low (n=6) |

| **Supplementary Table 4.**  *Summary of key extracted data.* | | | | | | |
| --- | --- | --- | --- | --- | --- | --- |
| **Study** | **Country of study** | **Study design** | **Community involvement** | **Study description** | **Autistic sample characteristics** | **Summary of findings relevant to the review** |
| Anderson et al. (2021) | United States | Qualitative | Not reported. | Unstructured interviews with 28 parents and 12 autistic young adults aiming to examine early employment-related expectations and experiences. Data were analysed using the constant comparative method. | *n* = 28; M age = 22.9 years; 79% male; 79% White; 32% with co-occurring intellectual disability; 54% employed | - Parents were concerned about their children working below their ability (i.e., being underemployed) - Some parents reported that their autistic child(ren) did not prioritise career progression (e.g., getting promoted/earning more money). - Parents were concerned about the lack of ‘career-building’ opportunities available to their child(ren) which limited career options. |
| Baldwin et al. (2014) | Australia | Quantitative | Not reported. | National survey of 130 autistic adults (‘high functioning’) examining whether overeducation is a prevailing issue for this group. Comparisons were made between the sample and the Australian Bureau of Statistics data on the general population. | *n* = 130; M age = 35.6 years; age range = 18 – 65 years; 68% male; 0% with co-occurring intellectual disability; 19% university educated; 100% employed | - 46% of the sample were considered overeducated |
| Balubaid (2017) | United Kingdom | Qualitative | Not reported. | Semi-structured interviews with 6 autistic adults in the UK and 13 parents of autistic people in Saudi Arabia, adopting a life history approach. Data were analysed using thematic analysis. | *n* = 6; M age = 32 years; age range = 18 – 51 years; 67% male; 0% employed | - Autistic participants highlighted a lack of support with progressing in their career. |
| Berman (2022) | United States | Qualitative and quantitative | Not reported. | Survey of 200 autistic people and ‘intensive’ interviews with 13 additional autistic people, gathering qualitative and quantitative data on self-determination and employment experiences/outcomes. Quantitative data were analysed using logistic regressions while qualitative data were analysed using inductive coding. | Quantitative:  *n* = 200; 46% male; 65% White  Qualitative:  *n* = 13 | - One participant said that their job coach had found them a job as a ‘dog poop scooper’ despite them having a Bachelor’s degree. - One participant also spoke of the lack of employment support for autistic people that are considered ‘high functioning’. The author suggested this may result in difficulties in getting ‘higher-quality jobs’. - Participants who highlighted positive aspects of the support they received noted the individualised nature of the supports, and explained how this helped them to achieve their own defined goals. |
| Braudis (2017) | United States | Qualitative | Not reported. | Five case studies of autistic people’s experiences of employment, gathered via interviews with the autistic people themselves, family members, employers and other relevant professionals. Transcripts were analysed against a theme codebook derived from existing literature, the interview questions, and interview data. | *n* = 5; 40% male; 100% employed | - 3 autistic interviewees discussed dissatisfaction in securing primarily low wage positions, 5 interviewees expressed concerns with the underemployment of autistic people, and 6 interviewees had concerns about low wages. - The parent of one autistic person said her son wasn’t interested in the ‘lower functioning jobs’ available. - Some interviewees were concerned about the social requirements of ‘upper level’ jobs and said this resulted in underemployment. - Two (non-autistic) interviewees raised concerns about autistic people having ‘unrealistic’ career expectations (e.g., expecting top-level positions) and/or having specific and unwavering career goals. - One autistic participant had an employment mentor which ‘contributed to his success at work’. |
| Brooke et al. (2018) | United States | Quantitative | Not reported. | A retrospective review of 139 records of autistic people that were referred to an employment support organisation. Aims to understand employment outcomes and job retention of those who achieved ‘competitive integrated employment’ via the organisation. | *n* = 104; M age = 26 years; age range = 19 – 66 years; 81% male; 60% White; 100% employed | - Over the 18-month evaluation period, 61 participants (87%) retained their job. Of those, 4 (~7%) were reported as advancing in their job (i.e., getting a new job in a higher position, gaining responsibilities, working more hours) |
| Buckley et al. (2021) | United Kingdom | Qualitative | Not reported. | Semi-structured interviews with 18 autistic people employed in the performing arts and 19 performing arts employers to understand their views and experiences of working in the performing arts, and occupational support. Data were analysed using reflexive thematic analysis. | *n* = 18; M age = 32.6 years; age range = 19 – 61 years; 50% male; 83% White; 17% with co-occurring intellectual disability; 100% employed | - Some participants were concerned that if disclosed to potential employers they would be ‘pigeon-holed into autism-specific work …perhaps limit their hiring opportunities’. - Some discussed difficulties with networking, and how to manage energy levels for networking. - When asked about the support they’d like to see more of, answers centred around wanting someone to consult (e.g., a mentor) about how to progress. |
| Buckley et al. (2022) | United Kingdom | Qualitative and quantitative | Not reported. | Randomised controlled trial examining the effect of professional mentoring on occupational self-efficacy of autistic people employed in the performing arts. Evaluation also included semi-structured interviews with the 15 autistic participants pre- and post- the intervention. Data were analysed using reflexive thematic analysis. | *n* = 15; M age = 32.6 years; age range = 19 – 54 years; 47% male; 87% White; 100% employed | - Many participants who signed up for the mentoring said they hoped for support in progressing further in their careers. - Mentees said the programme created new employment opportunities and supported them to map out career strategies. |
| Cheriyan et al. (2021) | United States, United Kingdom, 6 other countries not specified | Qualitative and quantitative | Not reported. | An online survey of 92 autistic and 774 non-autistic university students about their career aspirations, their perceived employment-related strengths, and obstacles they expect to encounter. Data were analysed using content analysis and compared using chi-square and/or logistic regressions. | *n* = 92; 53% male; 100% university students | - Autistic participants’ top goals in attending university were to improve their career prospects, followed by academic progression, interpersonal and personal development. |
| Cockayne (2019) | United Kingdom | Qualitative | Not reported. | Focus groups or individual interviews with 12 autistic people, 13 line-managers of autistic people, and 8 HR specialists. Focus groups/interviews concerned perceptions about being autistic in employment. Data were analysed using template analysis. | *n* = 21; 48% male; 57% White; 100% employed | - Some participants raised specific concerns about the 'people skills' typically required to take on higher-level positions. |
| Coleman & Adams (2018) | United States | Qualitative and quantitative | Yes – feedback on survey draft. | An online survey of 171 autistic people (or proxy reporters, e.g., parents) aiming to examine their vocational status, determine barriers to employment, evaluate government employment services, and determine possible methods to improve employment. Quantitative data were analysed descriptively, and qualitative data were ‘organised into different categories and summed’. | *n* = 171; table percentages exceeded 100% so unable to extract data | - Participants tended to have entry-level positions and worked an average of 25 hours a week, despite wanting to work 33 hours/week. - 11% of participants expressed a need for better job placement and 8% talked about being underemployed and/or being placed in jobs below their ability level. |
| Djela (2021) | United Kingdom | Qualitative | Not reported. | Qualitative review of an online consultation with 34 autistic people examining employment experiences. Data were analysed using ‘bottom-up coding’. | *n* = 34 | - 35% of participants said they experienced unfavourable treatment after disclosure, including invalidation, reduced career opportunities, disciplinary/dismissal procedures, bullying and isolation. |
| Dreaver et al. (2020) | Australia & Sweden | Qualitative | Yes – feedback on interview guides. | Semi-structured interviews with 23 company directors and/or line managers or autistic people about the organisational and individual factors facilitating the successful employment of autistic people. Data were analysed using ‘thematic analysis using a conventional content approach’. | *n* = 18; 83% male; 100% employed | - Line managers discussed the importance of providing opportunities for growth and advancement by “slowly building up the work role” and “figuring out how to challenge” their employees. |
| Elichaoff (2015) | United Kingdom | Qualitative | Not reported. | Semi-structured interviews with four (‘high functioning’) autistic people about their experiences of being an older autistic adult in the UK. Data were analysed using inductive thematic analysis. | *n* = 4; age range = 58 – 63 years; 50% male; 100% White; 100% university educated | - All participants were educated to at least degree level, yet all report a lack of career progression - One participant attributed lack of career progression directly to the fact they are autistic. |
| Grenawalt et al. (2020) | United States | Qualitative | Not reported. | A case study of 4 autistic employees, gathered via semi-structured interviews with 28 of their colleagues at HSM (a manufacturing plant employing autistic people through a private disability services provider). Data were analysed through a process of iterative coding. | *n* = 4; M age = 24.8 years; age range = 21 – 32 years; 100% employed | - Some autistic people had been given ‘more responsibilities’ following the initiative, and managers were ‘looking forward’ to giving them more responsibilities over time. |
| Ham et al. (2014) | United States | Qualitative | Not reported. | Two case studies of autistic young adults, highlighting the perceived success of their employment support. Unclear how the data were collected and/or analysed. | *n* = 2; M age = 23 years; age range = 23 years; 50% male; 100 % employed | - One of the participants had their working hours increased, after receiving the support outlined in their behaviour intervention and work productivity plan. |
| Harvery et al. (2021) | Australia | Qualitative and quantitative | Yes –priority setting, reviewing results and conclusions. | Survey of 149 autistic adults aiming to describe their employment profiles, with a specific focus on underemployment and underutilisation. Comparisons were made between the sample and the Australian Bureau of Statistics data on the general population. Logistic regressions were conducted to predict likelihood of underutilisation and underemployment. Qualitative data on workplace adjustments were analysed using the ‘coding reliability’ method. | *n* = 149; M age = 41.01 years; age range = 25 – 80 years; 40% male; 88% White; 3% with co-occurring intellectual disability; 61% university educated; 86% employed | - 37% of the sample were underemployed by either skill level or hours worked. - One participant said they had been turned down from an internal opportunity after disclosing their diagnoses and requesting workplace adjustments. - Autistic people with fewer autistic traits were more likely to be underutilised and underemployed. - The size of participants’ overall support networks predicted their odds of being appropriately employed. - Participants receiving workplace adjustments were 3.14 times more likely to be in a job adequately matched to their skill level, but this effect was not sustained when excluding self-identified autistic people. |
| Hayward et al. (2018) | Australia | Qualitative and quantitative | Yes – survey development and pilot testing. | Online survey of 46 autistic and 37 non-autistic people, examining whether employment experiences differ as a function of gender, diagnostic status, autistic traits, or age. Quantitative data were analysed using logistic regression. Qualitative data were analysed using inductive thematic analysis and compared between groups using chi-square or Fisher’s Exact Test. | *n* = 46; M age = 33.6 years; age range = 18 – 68 years; 39% male; 0% with co-occurring intellectual disability; 48% university educated | - One participant commented on the instability of their career, saying they had taken whatever was available to them. |
| Hayward et al. (2019) | Australia | Qualitative | Yes – survey development and pilot testing. | Online survey of 55 autistic people and 32 non-autistic people about their employment experiences. Analysis included inductive thematic analysis and thematic comparisons were made between the two groups. | *n* = 55; M age = 39.6 years; age range = 18 – 68 years; 42% male; 0% with co-occurring intellectual disability; 49% university educated; 76% employed | - One participant reported being ‘hopelessly overqualified’ for their job. - The authors state that ‘the resounding statement was that participants wished for an opportunity for growth and development.’ |
| Hedley et al. (2021) | Australia | Qualitative | Yes – focus group guide development | Semi-structured focus groups with 9 autistic people who took part in an autism employment program that provides 3-year employment opportunities for autistic people in information and computer technology (ICT). Analysis involved inductive data coding. | *n* = 9; M age = 23.97 years; age range = 19 – 29 years; 89% male; 44% university educated; 56% employed | - Autistic trainees were reluctant to disclose their diagnosis as they thought it ‘would detract from job prospects’. |
| Hurlbutt & Chalmers | United States | Qualitative | Not reported. | Six case studies of autistic people’s experiences of employment, gathered via interviews. Data were analysed using an ‘open coding procedure’ and field notes were taken to assist the process. | *n* = 149; M age = 31.83 years; age range = 25 – 65 years; 50% male; 17% with co-occurring intellectual disability; 67% university educated; 67% employed | - Participants had not been able to obtain jobs in the fields they had been trained in, and many had to take menial jobs (such as cleaning cat cages). - No participant had formal employment support but they generally said they would welcome such support, particularly in the form of a mentor. |
| Lindstrom et al. (2014) | United States | Qualitative | Not reported. | Four case studies of people with intellectual and/or developmental disabilities, describing their careers thus far. One case study was about an autistic man (Rick). Case studies were developed via interviews with the disabled people themselves, parents, high school teachers, adult agency personnel and/or current employers. Secondary data from a range of sources was gathered (e.g., questionnaires, observations, field notes, review of files). Data were analysed using a multi-stage qualitative analysis process. | *n* = 1; age = 27 years; male; White; with co-occurring intellectual disability; not university educated; employed | - Rick’s advocate described the “catch 22” of disability benefits, explaining he was unable to take on more work as he would lose his benefits. Yet, the authors suggested working in part-time service industry positions offered limited opportunities for pay increases or career advancement. |
| McLaren et al. (2017) | United States | Qualitative and quantitative | Not reported. | Description and evaluation of a pilot individual placement and support program for autistic people (adapted from a program for people with serious mental illness). Evaluation is presented in the form of 5 case studies, gathered via interviews with participants, their parents and employers, as well as secondary sources (e.g., questionnaires, review of medial records). Process for data analysis is unclear. | *n* = 5; M age = 22.2 years; age range = 19 – 28 years; 80% male; 0% with co-occurring intellectual disability; 0% university educated; 100% employed | - Each participant obtained an increase in pay and work hours, compared to previous employment, following the program. |
| Nicholas & Lau (2019) | United States | Qualitative | Not reported. | Semi-structured interviews with 4 autistic people employed with support of *Meticulon Consulting Inc* (a social enterprise that facilitates and supports employment in the information technology sector for autistic people), 9 business clients, and 3 employment support personnel. Data were analysed using content analysis. | *n* = 4; age range = 19 – 28 years; 100% male; 100% employed | - Employers identified the underrepresentation of autistic people in managerial roles within their organisations. - Employment support personnel spoke of how they gave autistic people opportunities to succeed” and reported that when well-supported, the autistic consultants were able to ‘thrive’. - Participants ) highlighted the need to match opportunities for career progression to autistic people’s individual career goals and support needs. |
| North (2021) | United Kingdom | Qualitative | Yes – research design | Focus groups with 15 autistic women about their experiences of employment. Data were analysed using thematic analysis. | *n* = 15; 0% male | - Participants often had one or more higher-level qualification and felt strongly they were capable of more. - Without the negative connotations associated with having a ‘condition’ or ‘disorder’, some participants she were able to hold high achieving, well paid roles and felt that the burden of being labelled autistic early in life could have held them back. - Job interviews and internal promotion processes involving interviews were discussed as barriers to progress in autistic women’s careers of choice. |
| Ortiz (2018) | United States | Qualitative | Not reported. | Focus group with 6 autistic people, and 2 individual interviews with a medical practitioner and an autistic self-advocate working as a consultant to organisational leaders. Focus groups/interviews concerned perceptions about the most beneficial support for autistic people in an employment context. Data were analysed using in vivo coding and analytic memos. | *n* = 6; age range = 18 – 35 years; 83% male; 83% White; 0% with co-occurring intellectual disability | - In one anecdote, an employee’s representative had suggested she could work within a factory putting Velcro straps together, despite having a Masters degree. |
| Raymaker et al. (2023) | United States | Qualitative | Yes – priority setting, development of research materials, data analysis and subsequent recommendations. Several members of the research team were autistic. | Semi-structured interviews with 45 autistic employees/job seekers, and 11 supervisors/support professionals about their experiences of ‘skilled employment’ and employment success. Data were analysed using inductive thematic analysis. | *n* = 45; M age = 36 years; age range = 21 – 65 years; 40% male; 78% White; 73% university educated | - When asked about what successful employment means, answers converged around opportunities for professional growth, good work/life balance, financial independence, sense of community at work, feeling valued, doing meaningful work, and being a part of an accepting work culture. - A number of participants discussed their experiences with employment service and support systems, with many describing how such services struggled to understand and connect them with skilled settings. - One participant highlighted burnout as a key contributor to a lack of long-term employment success for autistic people. |
| Romualdez (2021) | United Kingdom | Qualitative and quantitative | Not reported. | Study 1 (chapter 2):  An online survey of 238 autistic people, gathering qualitative and quantitative data about their experiences of disclosing an autism diagnosis at work. Quantitative data were analysed descriptively and qualitative data were analysed using content analysis. | *n* = 238; 33% male; 95% White; 71% university educated; 66% employed | - Participants reported concerns about disclosing their autism diagnosis through fear it may be used as an excuse not to promote them. |
| Sang et al. (2022) | United Kingdom | Qualitative | Yes – lead author is a disabled academic. | Semi-structured interviews with 75 disabled academics about their employment experiences. Data for two autistic participants (Ainsley & Tina) could be extracted. Data were analysed using inductive and deductive coding in iterative cycles. | *n* = 2; 0% male; 100% employed | - One autistic participant explained that they were unable to work full-time, which they felt would inevitably have a negative impact on their career. |
| Sharpe et al. (2022) | Australia | Qualitative | Not reported. | Semi-structured interviews with 9 autistic people about their experiences of gaining and maintaining mainstream employment. Data were analysed using thematic analysis. | *n* = 9; M age = 22.5 years; age range = 22 – 32 years; 78% male; 0% university educated; 100% employed | - 4 participants reported future career aspirations that did not match their current role. - Although participants raised future aspirations, some felt they didn’t have the skills or abilities to meet their career aspirations. |
| Wehmen et al. (2017) | United States | Quantitative | Not reported. | Randomised clinical trial examining the effect of Project SEARCH + Autism Spectrum Disorder Supports on employment outcomes. Data were compared using chi-square and independent t-tests, as well as generalised estimating equations and mixed repeated measures. | *n* = 49; M age = 19.5 years; age range = 19 – 21 years; 71% male; 51% White | - Wages had significantly increased over time for the treatment group, but this didn’t remain significant when unemployed participants were removed from the analyses - Number of hours worked also significantly increased over time for the treatment group, but this didn’t remain significant when unemployed participants were removed from the analyses - At the 12-month follow-up, there was a significant difference in hours worked between treatment and control groups, but this didn’t remain significant when unemployed participants were removed from the analyses. |
| Wong et al. (2021) | United States | Qualitative | Not reported. | Semi-structured interviews with 20 school personnel working with autistic students about their experiences of preparing autistic youth for employment. Data were analysed using thematic analysis. | N/A | - School personnel viewed autistic youth as having unrealistic career goals and difficulty setting practical career goals. |
| Wood & Happé (2021) | United Kingdom | Qualitative | Yes – survey development | Online survey of 149 autistic people who were currently working, or had previously worked, in the education sector, gathering information about their employment experiences. Data were analysed thematically using a process of constant comparison. | *n* = 149; age range = 19 – 62 years; 18% male | - A former teacher said that one school where she had worked had asked her not to inform parents that she was autistic, a factor that had contributed to her career being ‘in ruins’. The same teacher wrote that she is now unemployed and ‘relying on food banks to survive’. - An early years practitioner stated that she was ‘sacked after diagnosis’. - The data indicated a significant correlation between the lack of support and mental health issues, leading to high levels of fatigue and burnout. Some participants had left the education profession due to these issues, though, some of those still working in schools currently were experiencing impediments to their work and career progression as a result of their continuing mental health issues. |
